# Identify novel elements of knowledge with word embedding

**DOI:** 10.1371/journal.pone.0284567

**Published:** 2023-06-20

**Authors:** Deyun Yin, Zhao Wu, Kazuki Yokota, Kuniko Matsumoto, Sotaro Shibayama

**Affiliations:** 1 School of Economics and Management, Harbin Institute of Technology (Shenzhen), Shenzhen, China; 2 World Intellectual Property Organization, Geneva, Switzerland; 3 School of Business Administration, Hitotsubashi University, Tokyo, Japan; 4 National Institute of Science and Technology Policy, Tokyo, Japan; 5 CIRCLE, Lund University, Lund, Sweden; 6 Institute for Future Initiative, The University of Tokyo, Tokyo, Japan; University of Naples Federico II: Universita degli Studi di Napoli Federico II, ITALY

## Abstract

As novelty is a core value in science, a reliable approach to measuring the novelty of scientific documents is critical. Previous novelty measures however had a few limitations. First, the majority of previous measures are based on recombinant novelty concept, attempting to identify a novel combination of knowledge elements, but insufficient effort has been made to identify a novel element itself (*element novelty*). Second, most previous measures are not validated, and it is unclear what aspect of newness is measured. Third, some of the previous measures can be computed only in certain scientific fields for technical constraints. This study thus aims to provide a validated and field-universal approach to computing element novelty. We drew on machine learning to develop a word embedding model, which allows us to extract semantic information from text data. Our validation analyses suggest that our word embedding model does convey semantic information. Based on the trained word embedding, we quantified the element novelty of a document by measuring its distance from the rest of the document universe. We then carried out a questionnaire survey to obtain self-reported novelty scores from 800 scientists. We found that our element novelty measure is significantly correlated with self-reported novelty in terms of discovering and identifying new phenomena, substances, molecules, etc. and that this correlation is observed across different scientific fields.

## 1. Introduction

Novelty constitutes a core value in science [1–6]. Novelty serves as an important criterion for the assessment of scientific performance and for decision makings such as employment and funding allocation [1, 7–9]. It is thus critical that scientific novelty be reliably measured. Several bibliometric indicators for novelty have been proposed [10–16], but there remain a few fundamental limitations.

First, most previous measures have not been validated. As novelty is a multifaceted concept, it is not entirely clear what aspect of novelty is captured or even whether novelty is measured at all. Second, the dominant approach of measuring novelty in previous studies appears to emphasize a specific type of novelty at the cost of overlooking other types. Namely, previous approaches mostly are based on the concept of *recombinant* novelty [17–19], with which a scientific document is considered novel if it includes a new or rare combination of knowledge elements (e.g., keywords and cited references) [12, 14, 15, 20, 21]. In contrast, few studies address the newness of a knowledge element itself [11, 22, 23]. This is intriguing as the novelty of an element ("element novelty" hereafter), which serves as a building block of recombinant novelty, is conceptually and practically more fundamental [11, 23, 24]. Third, due to technical constraints, some of the previous measures can only be computed in certain scientific fields, which is a critical flaw when the boundary of disciplines becomes increasingly ambiguous [25, 26]. This study thus aims to develop a validated and field-universal approach to computing scientific novelty based on the element novelty concept.

Element novelty being underserved is attributed to a few technical challenges. One simple approach to measure element novelty is to draw on text information and consider a document to be novel if it includes a new word [23]. However, this approach faces a challenge when a document includes a new word that is merely a synonym of or similar to an existing word. Such an issue can be addressed by constructing controlled vocabulary dictionaries, but it requires substantial manual work and such efforts tend to be constrained within specific expertise areas.

To overcome these challenges, we drew on a natural language processing (NLP) technique to develop a word embedding model that covers all scientific fields. The technique extracts the semantic information of text data and maps each word onto a high-dimensional vector space [27], which functions as a vocabulary dictionary. After validating that our word embedding model does capture semantic information, we apply the model to measure element novelty. Word embeddings allow us to quantify the distance between a pair of text data. Thus, we can measure distances between documents and thereby identify a *novel* document that is distant from other documents. To validate this novelty measure, we carried out a questionnaire survey, asking the authors of sampled scientific articles to self-evaluate multiple novelty aspects of the articles they authored. We confirm that our novelty measure is correlated with the self-reported novelty of a knowledge element in various scientific fields. We further compare our novelty measure with a few previous novelty measures and demonstrate the advantage of our approach.

The contribution of this study is twofold. First, this is the first to provide a validated and field-universal approach to computing element novelty. Second, our word embedding model offers a technical basis to interpret scientific text information, which can be applied not only for calculating novelty but also for describing other qualities of knowledge such as the diversity of a knowledge portfolio and the temporal evolution of knowledge [28].

This paper is structured as follows. Section 2 reviews previous novelty measures and technical backgrounds to compute novelty. Section 3 outlines the construction of the word embedding model and validates the model. Section 4 explains the operationalization of the novelty measure based on the word embedding and presents the result of validation analyses. Section 5 summarizes the results and conclude.

## 2. Related works

### 2.1 Measures of novelty

Novelty is one of the core values in science as it drives the progress of science fundamentally [1–4, 28]. Scientists can make novel contributions in various manners, such as proposing a new theory, discovering a new phenomenon, and developing a new method. For example, Guetzkow et al. [29] identified various dimensions of novelty (approach, theory, method, data, and findings) based on in-depth interviews of social scientists and suggested that relevant novelty dimensions can differ across scientific fields. Dirk [30] conducted a questionnaire survey of life scientists and found that the respondents considered novelty in hypotheses more important than novelty in methods.

Given the multi-dimensionality of novelty, it is critical to understand what novelty aspect is captured by bibliometric novelty indicators, which has been insufficient due to limited validation efforts with a few exceptions [31, 32]. Nonetheless, previous measures are broadly categorized into two groups–recombinant novelty and element novelty–and thus might capture two aspects of novelty.

#### 2.1.1 Recombinant novelty

Recombination has been considered as a source of novelty widely in the literature. The creativity literature contends that linking remote elements is a path to creativity [12, 17, 18]. The management literature suggests that a novel invention results from a synthesis of diversified ideas [19, 33–35]. To operationalize this novelty concept, previous measures consider a document to be novel if it includes a new or rare combination of knowledge elements, where a knowledge element is operationalized differently.

The most straightforward operationalization is to use a word as a knowledge element. For example, Boudreau et al. [13] measured the novelty of a grant proposal based on a new combination of Medical Subject Heading (MeSH) keywords. Similarly, Zhang et al. [36] measured the novelty of a scientific paper based on a new combination of keywords that appeared in the abstract. These approaches are intuitive, but the ambiguity of text information (e.g., synonyms) is a concern, which could be sorted out by a controlled vocabulary dictionary.

The most widely used approach is to consider a cited *journal* as a knowledge element [12, 15, 21, 28]. A document is considered novel if it cites documents in two journals that have rarely been cited together. Citation implies that knowledge in a cited document is used by a citing document [37]. Thus, a document citing a rare journal pair implies that a rare pair of knowledge is integrated. However, since a journal is a highly aggregated unit as a knowledge element, its validity is under dispute despite practical merits [25, 32].

The third operationalization is to use a cited *reference* as a knowledge element instead of a journal. A document is considered novel if it cites a set of documents that have rarely been cited together. Dahlin et al. [20] proposed an indicator for the novelty of patents based on a rare combination of cited patents. Trapido [14] applied this approach to scientific documents in the field of electrical engineering, and Matsumoto et al. [31] further extended it to all fields. These operationalizations draw on citation network analysis to assess the newness of a reference combination.

#### 2.1.2 Element novelty

The key limitation of recombinant novelty is that it overlooks the novelty of a knowledge element itself. Radically new ideas often have no discernible antecedent, and fundamental breakthroughs often result from exploring an uncharted knowledge space [24]. Such isolated incidents of novelty may not be captured by the recombinant novelty measures. In line with this notion, Ahuja et al. [24] and Trajtenberg et al. [38] measured the novelty of patents by considering a patent that has no reference to be novel, though this operationalization is not applicable to scientific documents.

A more generally applicable approach is to draw on the first appearance of a word(s) in a document. If a document includes a certain word or a sequence of words (N-gram) that is new to the world (e.g., the name of a previously unknown chemical compound), it implies that the document delivers novel information. For example, Chen et al. [11] and Packalen et al. [22] use the first appearance of n-gram in medical papers to measure the novelty of a scientific documents. Balsmeier et al. [39] and Arts et al. [40] also assess the novelty of a patent based on whether it refers to a certain word for the first time. Though this is intuitive, the newness of a word can be misleading as it overlooks synonyms and similar words.

A solution to this problem is to draw on controlled vocabulary dictionaries. Azoulay et al. [23] computed the average age of MeSH keywords to evaluate the novelty of an article. Similarly, Strumsky et al. [41] measured the originality of patents by the appearance of new patent classes. These are nonetheless an imperfect solution as building dictionaries takes substantial expert effort and existing dictionaries are often field-specific.

### 2.2 Word embedding

The word embedding technique addresses the limitations in the above-mentioned approaches. Word embedding is a technique to project a word into a high-dimensional vector space. It computes a vector for each word based on the co-occurrences of words in a text corpus [27]. By inferring the meaning of words from the context surrounding the word, the technique captures semantic relationships from unstructured text data and encodes it in a concise expression. It also reduces noises caused by synonyms and disciplinary jargons. Furthermore, a word embedding model can be extended to multiple languages. Importantly, the process of training word embedding models requires little human intervention or expert knowledge. Though training a model takes computer resources, such trained models can be repeatedly used. Thus, a word embedding model serves as a machine-generated vocabulary dictionary. The word embedding technique is gaining popularity as the performance of machine learning has been improving. In fact, word embeddings have been applied to scientific text for various purposes. For example, Tshitoyan et al. [42] captured the structure of extant knowledge in material sciences with word embeddings and predicted future discoveries in the field.

A few studies have used word embeddings to measure novelty. As a word embedding model assigns a vector to each word, one can quantify the distance between any pair of words. By aggregating word vectors at the document level, semantic information included in a document can be expressed as a vector, and thus, the distance between a pair of documents can be computed. This offers the technical basis for measuring novelty. A measure of recombinant novelty based on word embeddings was proposed by Shibayama et al. [16], in which the distance between cited references and thus the newness of a reference combination is computed. Shibayama et al. [16] drew on an existing word embedding model in biomedicine (*scispaCy*), and its applicability to other fields has not been examined. Hain et al. [43] similarly used the word embedding technique to measure distances between patent documents, and operationalized novelty by the average distance between a focal patent and a set of previous patents. This serves as a measure of element novelty in which a patent document itself is considered as the unit of knowledge element. Unfortunately, the validity of this measure is not examined.

Some pre-trained word embedding models are publicly accessible, such as *scispaCy* in biomedicine [44–46], *mat2vec* in materials sciences [42, 47] and *ES2Vec* in earth science [48], but no word embedding trained on field-unspecific scientific vocabularies is publicly available yet. To the best of our knowledge, the current study is the first to build a word embedding model for the whole universe of scientific text to measure element novelty and test its validity.

## 3. Developing word embedding model

### 3.1 Training word embedding model

To train a word-embedding model for scientific text, we collected all scientific publications in English in the Science Citation Index Expanded (SCIE) from the Web of Science (WoS) database. The database includes publications from all fields except for social sciences and humanities. We drew on 23 million (23,350,859) records with both titles and abstracts published between 1998 and 2018. We focused on titles and abstracts rather than full text for greater accessibility and for conciseness and efficiency in computation, although full text might provide more detailed and comprehensive information.

For each publication, we combined the title text and the abstract text and carried out a series of cleaning procedures with the Python library–Natural Language Toolkit (NLTK). Specifically, we lowercased the text, removed words consisting purely of numbers and special symbols, and stemmed each word. To further improve the computing efficiency and avoid the noise from trivial or misspelled words, we also removed words that appeared less than five times in the entire database. Using this cleaned text of the 23 million documents, we trained their word embeddings with the *word2vec* model through Python’s Gensim package (https://radimrehurek.com/gensim/). Word2vec reduces the high dimensionality of the word space by training a neural network model while preserving semantic relationships between words. The trained model assigns to each word a "word embedding", which is a vector typically in several hundred dimensions. In training our word embedding model, we set default values for most parameters. Among others, we set the vector size as 300 following the original study of *word2vec* [27]. Finally, we obtained 300-dimensional word vectors for 3,208,589 unique words. The median number of distinct words in a document is 127.

To examine the coverage of the words, we compared them with WoS keywords and found that 76% of WoS keywords are included in our model. Thus, a quarter of WoS keywords are not included possibly because they are used only infrequently and thus dropped from our model training.

### 3.2 Validating word distance

Before developing the novelty measure, we test whether our word embedding model conveys semantic information of a word. Specifically, we examine whether our model properly represents the distance between words–i.e., related words have similar vectors and unrelated words have remote vectors. For this test, we draw on an extant word embedding model, *scispaCy* (*en_core_sci_lg*: https://github.com/allenai/scispacy). *ScispaCy* is an established and publicly available word embedding model, offering vector representations of 600,000 vocabularies specializing in biomedical texts [44]. Shibayama et al. [16] used *scispaCy* to develop a measure of recombinant novelty and validated it with survey data, which gives confidence in the performance of the model. Here, we randomly sampled 5,000 words common to *scispaCy* and to our word embedding model and made all possible pairs of the 5,000 words. For each word pair, we compute 1) the cosine distance based on our word embedding and 2) the cosine distance based on *scispaCy* word embedding. Finally, we assessed the correlation between the two distance scores, finding a strongly significant correlation (*r* = .411, p < .001). This suggests that two words that are considered (dis)similar in *scispaCy* are also considered (dis)similar in our model, supporting the validity of our word embedding model.

### 3.3 Validating document distance

Next, we test whether our word embedding model can measure distances between documents by aggregating word vectors. For this analysis, we randomly sampled 1,000 pairs of research articles in five broad disciplines (biology, chemistry, medicine, engineering, and physics) respectively, in which both articles in each pair are published in the focal field in 2018. For each pair, we compute their distance in two approaches–one based on our word embeddings and the other based on a previously established approach–and assess if they are sufficiently correlated.

We first compute the document distance based on our word embedding model by aggregating the word vectors at the document level. For simplicity, we took the mean of the vectors of all words included in the title and abstract of a focal document. Then, we computed the cosine distance of the vectors of two documents.

Second, as a previously established approach, we drew on co-citation information of a document pair [14, 31]. Namely, the distance between a pair of documents *i* and *j* is given by:

$$1-\frac{{coref}_{ij}}{\sqrt{{ref}_{i}∙{ref}_{j}}}$$ (1)

where *ref*_*i*_ is the number of references cited by *i* and *coref*_*ij*_ is the number of references cited by both *i* and *j*. This is based on the assumption that a pair of documents are similar if they cite a similar set of documents. In sampling document pairs we included only those with at least one co-cited reference. If we had sampled document pairs completely randomly, it would be highly unlikely that they share any reference. Since the co-citation distance is not a perfect measure of document distance, we expect only a modest correlation between the two distance scores. We did find a significant correlation (*r* = .22, p < .001), suggesting that our word embedding model conveys semantic information at the document level.

#### 3.3.1 Field difference

**Fig 1:**
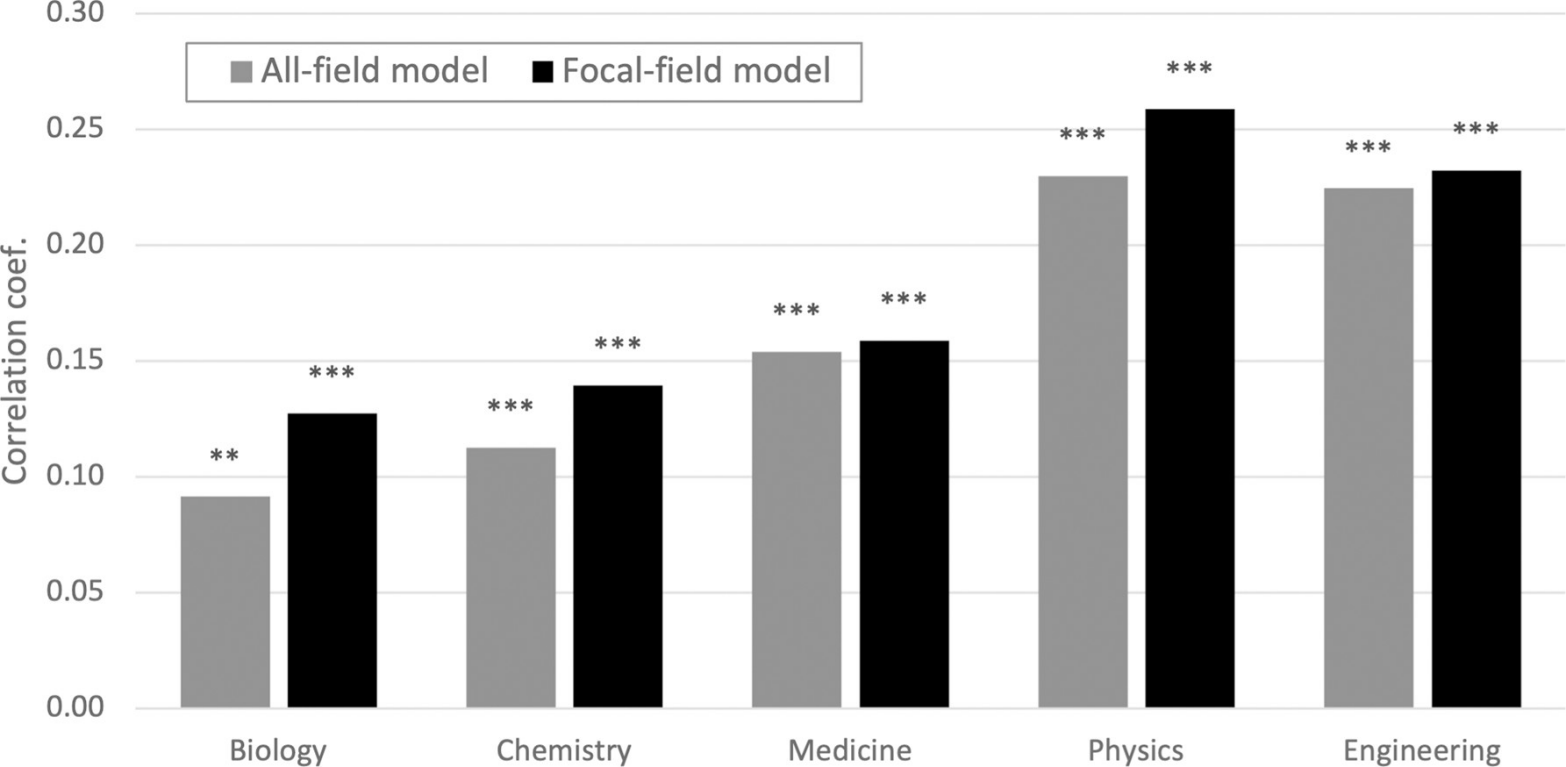
Document distance by scientific field. Pearson’s correlation coefficient with the co-citation distance. N = 1,000. Gray bars: based on the word embedding trained by text in all fields. Black bars: based on the word embedding trained by text in the focal field. **p<0.01.***p<0.001.

It is plausible that the validity of the word embedding model differs across scientific fields due to potential differences in the nature of knowledge. We thus split the sample into five fields and found some differences in the magnitude of correlations (Fig 1). Relatively higher correlations are found in physics (*r* = .23) and engineering (*r* = .22) while lower correlations are found in chemistry (*r* = .089) and biology (*r* = .084) (light bars).

The difference across fields may also be attributable to a bias in the training data. That is, our training data include more publications in some fields (e.g., medicine) and less in other fields, which may affect the performance of the word embedding model. To investigate this possibility, we also trained five sets of word embedding models with text data only in a certain field. We then replicate the correlation analysis in each field by computing document distance based on the word embedding trained with the text data in the focal field (dark bars). In fact, the correlation coefficients become larger with the field-specific model in all fields except for medicine. This suggests that the performance of the word embedding technique may be improved by limiting the scope of knowledge areas.

#### 3.3.2 Time difference

**Fig 2:**
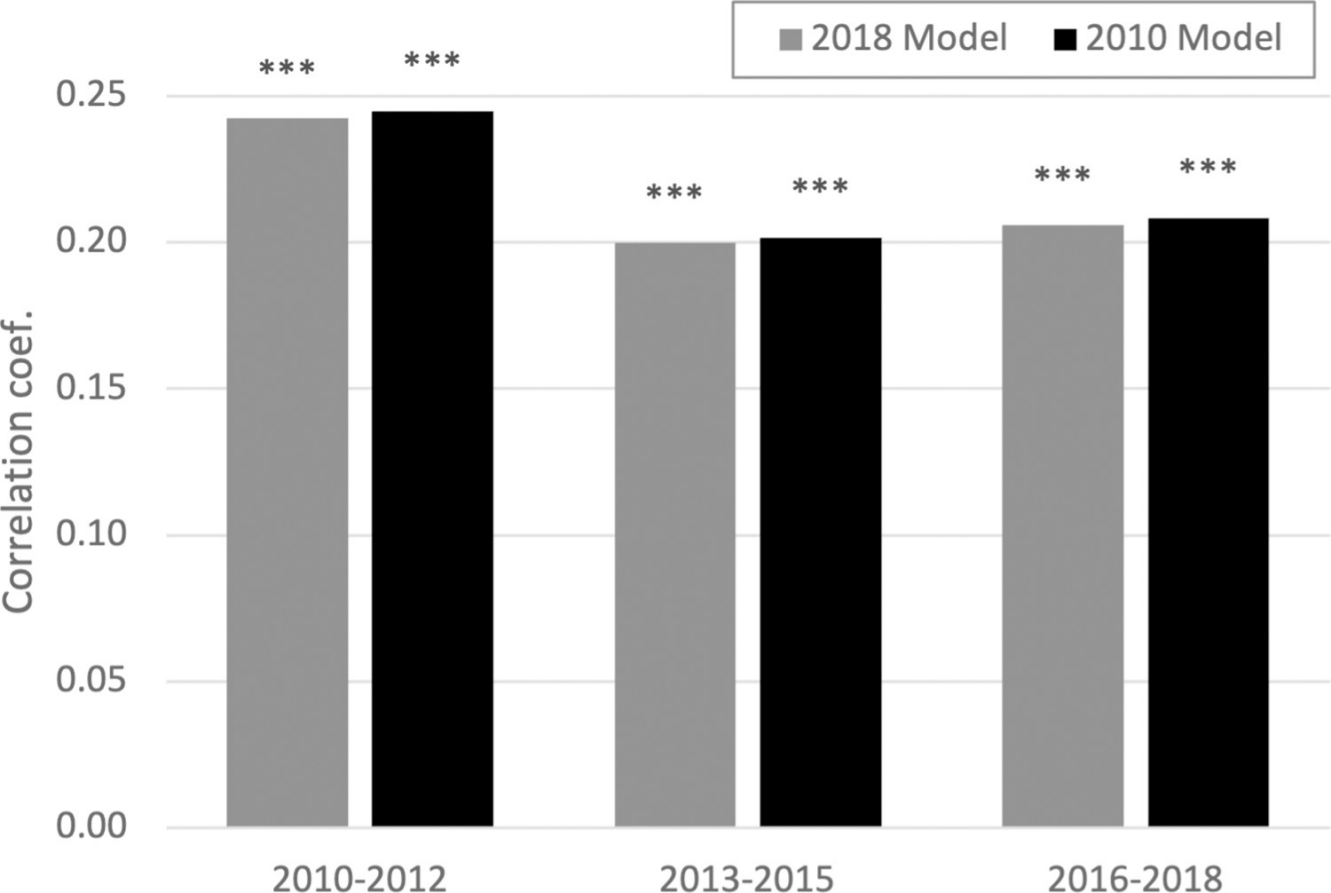
Document distance over time. Pearson’s correlation coefficient with the co-citation distance. N = 3,000. Gray bars: based on the word embedding trained by text published until 2018. Black bars: based on the word embedding trained by text published until 2010. ***p<0.001.

In the main analysis, the training data for modelling the word embedding include publications up to 2018, and the test data are publications in 2018. We then investigated how the temporal gap between training data and test data influences the measurement of documents’ distance (Fig 2). First, we applied our model to older documents (light bars). Namely, we sampled 3,000 document pairs in three 3-year time windows (2010–12, 2013–15, and 2016–18) and replicated the correlation analysis. If the evolution of knowledge is fast, distances between older documents can be measured less effectively. To the contrary, if the knowledge structure is stable over time, distances between older documents can be measured more precisely because words in older documents have a longer history of use and thus the model is better trained. The result showed greater correlation coefficients in the 2010–12 window than in the 2016–18 window, which appears consistent with the latter argument. Next, to investigate whether the word embedding model can be applied for future documents, we developed another word embedding model with training data up to 2010. Then, we apply this model to the same three windows of document pairs (dark bars). The result shows lower correlation coefficients in more recent years, probably because words in more recent documents are less likely to be included in the training data.

## 4. Measuring novelty

### 4.1 Operationalization

Based on the word embedding, we computed a series of element novelty measures in the following steps. First, we assigned a vector to each document by taking the mean of the word vectors of all words included in the title and abstract text of the document. The document vector of document *i* is given by

$$v_{i}=\frac{1}{w_{i}}\sum_{k=1}^{w_{i}}v_{ik}$$ (2)

where *v*_*ik*_∈ℝ^300^ is a word vector of the *k*-th word and *w*_*i*_ is the number of words included in document *i*.

Second, for each focal document *i*, we computed its distance from all other documents published before document *i*. Suppose that document *i* has *N*_*i*_ documents previously published. For each of the *N*_*i*_ documents, denoted by *j*∈{1,…,*N*_*i*_}, we computed the cosine distance given by:

$$d_{ij}=1-\frac{v_{i}∙v_{j}}{\left|v_{i}||v_{j}\right|}$$ (3)

The cosine distance ranges from 0 to 2 with a larger value indicating a larger distance.

Finally, we computed the element novelty of each document. We sort all distance scores *d*_*ij*_ and use the *q*-percentile values as novelty scores (*ElementNovel*_*q*_), where the 100-percentile is the maximum and the 0-percentile is the minimum. The intuition is that a document is considered novel if it is distant from other documents. Even if the minimum distance is still high, it indicates that the document is distant from all other documents. Therefore, low percentile values are of our particular interest. We computed a series of scores (*q*∈[0,100]) but mainly use the lower-percentile scores (*q* = 0, 1, 5, 10, …).

### 4.2 Questionnaire survey for validation

To test the validity of the proposed novelty measure, we conducted a questionnaire survey and draw on self-reported novelty scores. We sampled the respondents in the following steps. First, we identified contact authors whose email addresses are available in WoS publication records. We then selected contact authors who had published at least four English-written papers ("article", "letter", or "proceedings paper"). This is to ensure that the authors have sufficient research experience and can reasonably assess novelty. For each author, we selected one paper that was most recently published (as of 2018). To avoid recall bias we dropped authors whose latest paper was published in 2015 or before. Finally, we randomly sampled 10,357 author-paper pairs. After three waves requests, 1,020 were bounced back and 825 responses were collected (response rate = 8.8%). The scientific fields of the papers are biology (28%), chemistry (28%), medicine (13%), engineering (13%), physics (14%), and other fields (5%). We found no significant response bias in terms of scientific fields.

**Fig 3:**
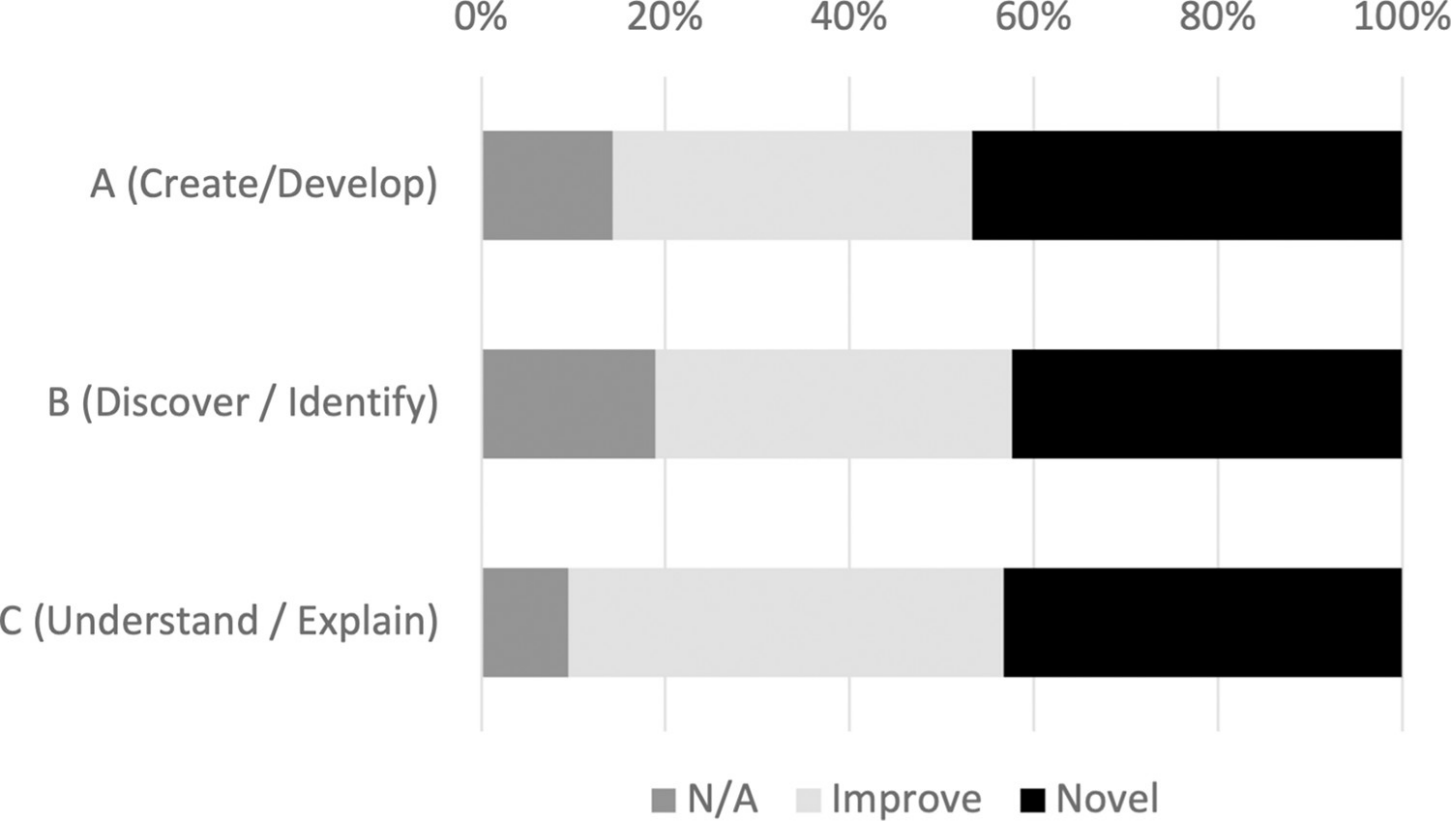
Survey scores of novelty.

The survey inquired into various qualities of scientific papers, of which this study draws on one section including three items concerning scientific novelty. We developed the questionnaire items partly informed by previous surveys on scientific novelty [29, 30] and tested them with a small-scale pilot survey. The items assess how the respondent’s selected paper make contribution in three aspects: A) in creating/developing something (e.g., methods, materials, data, chemicals), B) in discovering/identifying something (e.g., phenomena, substances, molecules), and C) in understanding/explaining something (e.g., mechanisms, theories). Each aspect is assessed on a 3-point scale–"not applicable" (previously reported or irrelevant), "improved" (improvement over previously reported findings), and "novel" (not previously reported) (Fig 3). For the following validation we mainly use three dummy variables (*Novel*_*A*_, *Novel*_*B*_ and, *Novel*_*C*_), each assigned 1 if the response is "novel" and 0 otherwise. We also use 3-point ordinal variables S1 Fig.

### 4.3 Validating novelty measures

**Fig 4:**
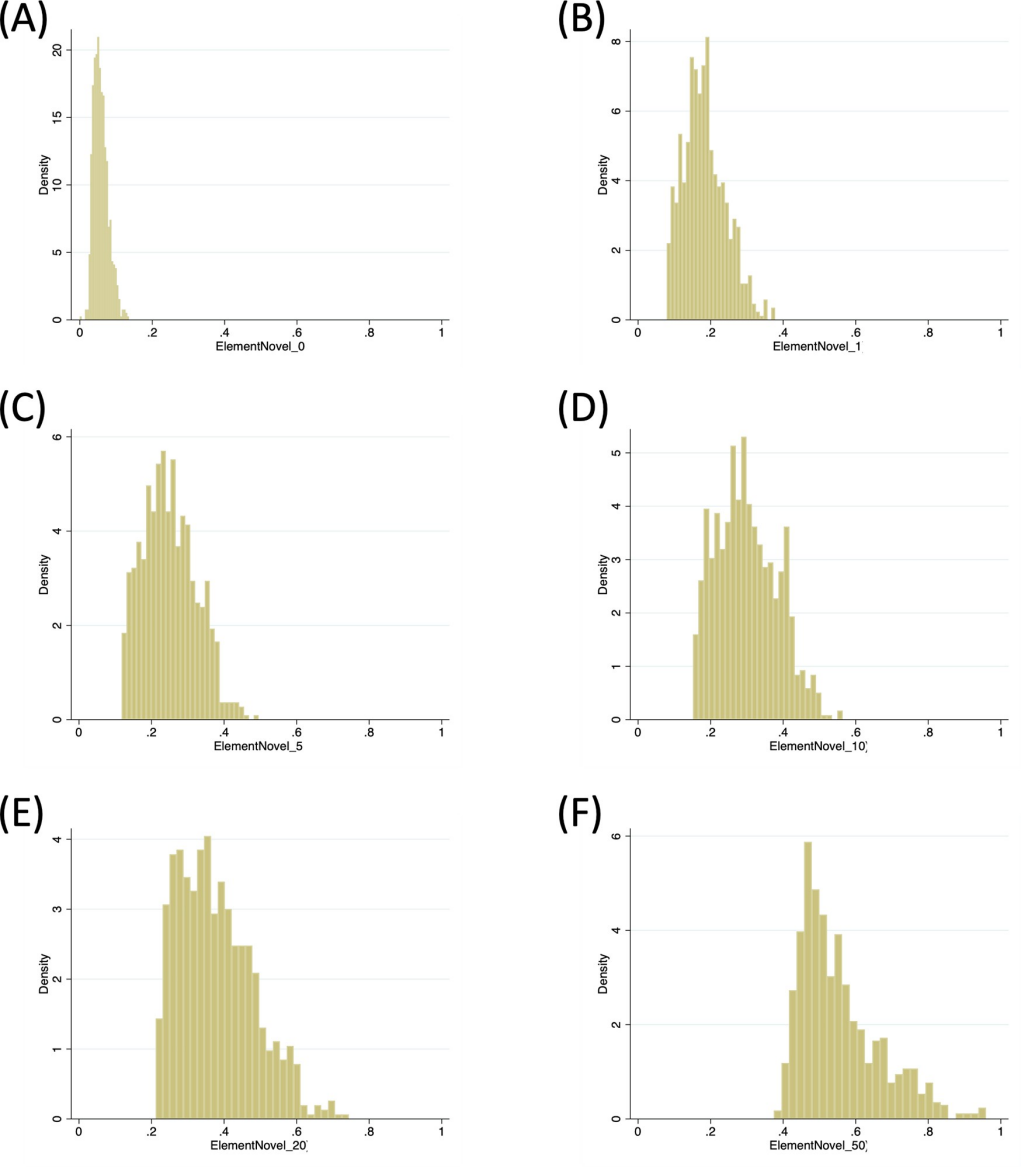
Distribution of element novelty. The histogram of element novelty measures with various *q*’s (A: 0, B: 1, C: 5, D: 10, E: 20, and F: 50).

Fig 4 illustrates the histograms of element novelty measures (*q* = 0, 1, 5, 10, 20, and 50) for the survey respondents’ papers. All measures are well distributed though somewhat right-skewed.

**Fig 5:**
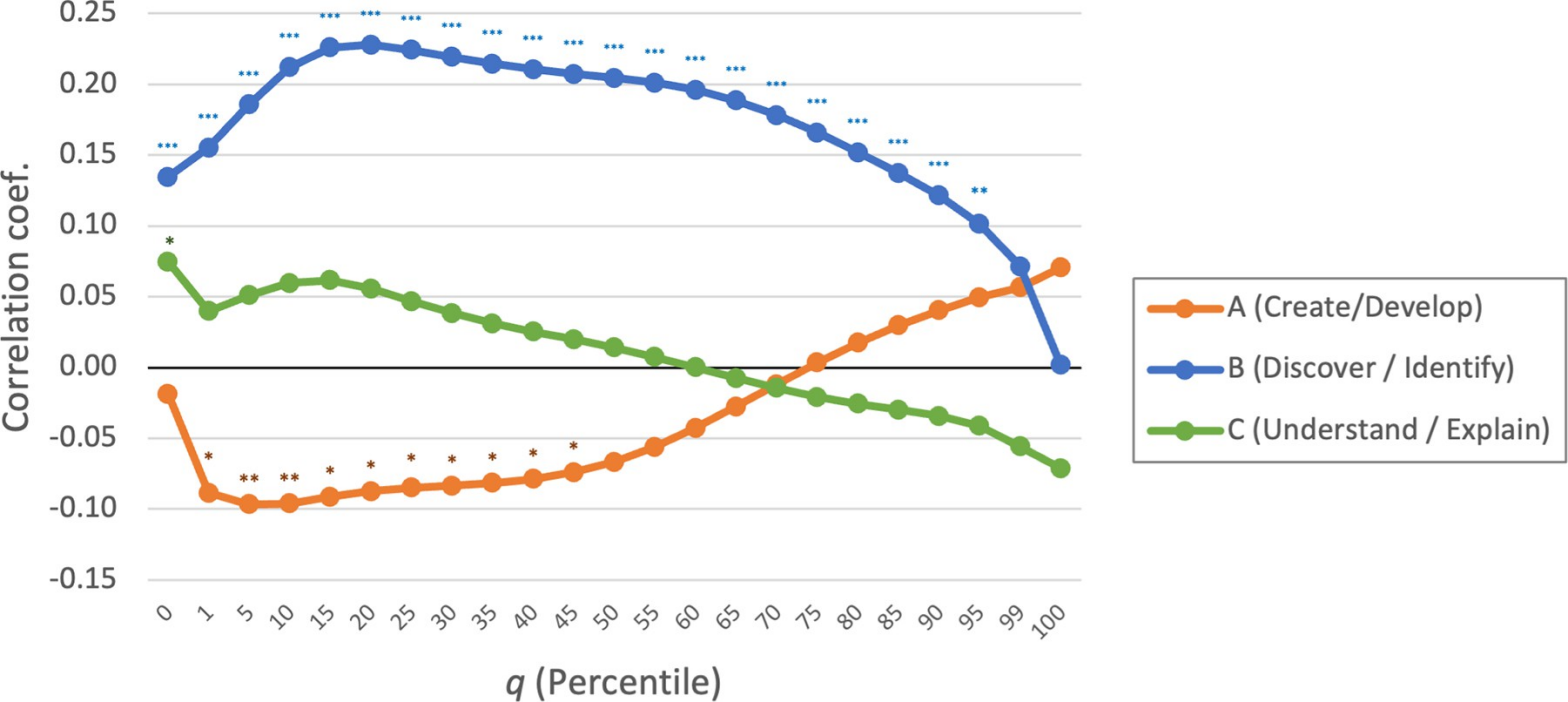
Correlation between element novelty scores and survey scores. N = 754. *p<0.05. **p<0.01.***p<0.001.

Fig 5 plots the correlation coefficients between element novelty measures and the survey novelty scores in three aspects. Although it presents the whole range (*q =* [0,100]) of element novelty measures, element novelty in lower *q* values is of our interest. The result indicates significant correlation between our element novelty measure and *Novel*_*B*_ survey score ("discovering and identifying"). This is consistent with our expectation in that "discovery" and "identification" (e.g., phenomena, substances, molecules) tend to be represented by a word or relatively short expressions–i.e., literally elemental. In contrast, correlations with *Novel*_*C*_ ("understanding and explaining") are largely insignificant. This is also understandable in that "understanding" and "explanation" (e.g., mechanisms, theories) are likely to be composite knowledge and less easy to capture in an isolated element. Interestingly, correlations with *Novel*_*A*_ ("creating and developing methods, materials, data, chemicals") are negatively significant, though rather modestly.

**Fig 6:**
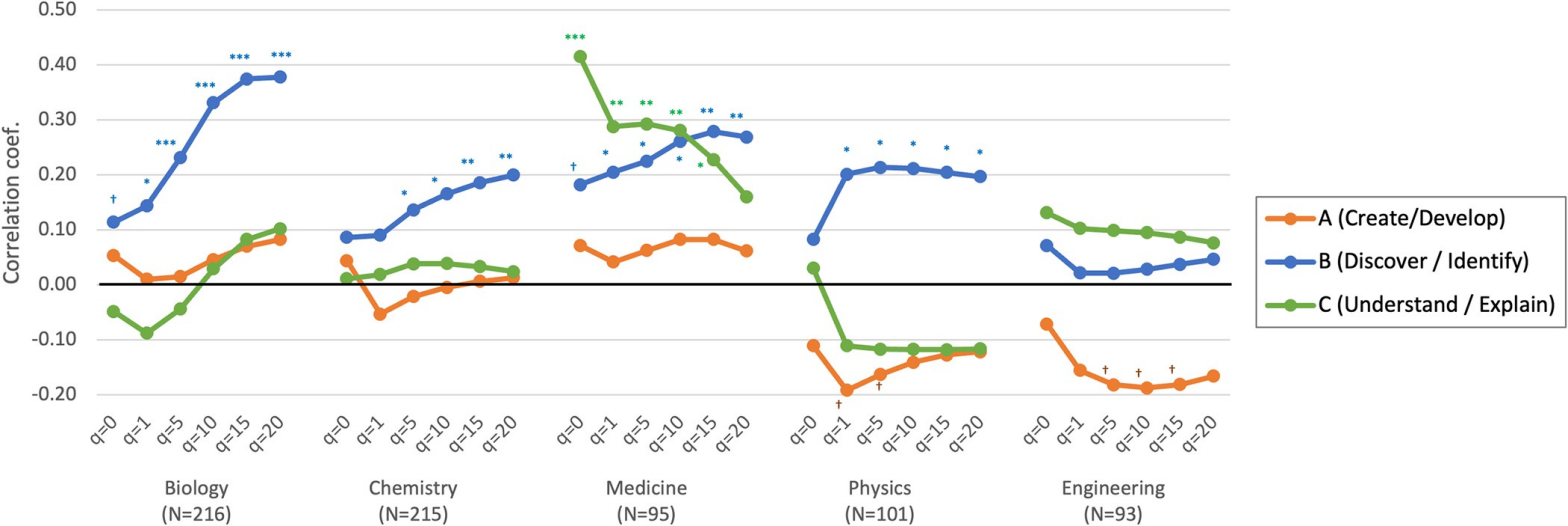
Correlation between element novelty scores and survey scores (field breakdown). A series of element novelty scores (*q* = 0, 1, 5, 10, 15, and 20) are displayed. ^†^p<0.1. *p<0.05. **p<0.01.***p<0.001.

As the nature of novelty may differ across fields [49], we split this correlation analysis into five fields (Fig 6), finding somewhat different patterns between fields. First, the significant correlation with *Novel*_*B*_ ("discovering and identifying") is stable in all fields except engineering. S1 Fig, nonetheless, shows modest correlations between *Novel*_*B*_ and 3-point survey scores of novelty in engineering. Thus, our novelty measure seems to be associated with elemental advancement in all fields. Second, *Novel*_*C*_ ("understanding and explaining") is largely insignificant except in medicine. This may imply that new mechanisms and theories in medicine tend to be expressed in simpler terms. Third, *Novel*_*A*_ ("creating and developing") is found significantly negatively correlated in engineering and physics. This implies that new methods and materials in these fields tend to be closely related to existing knowledge.

**Fig 7:**
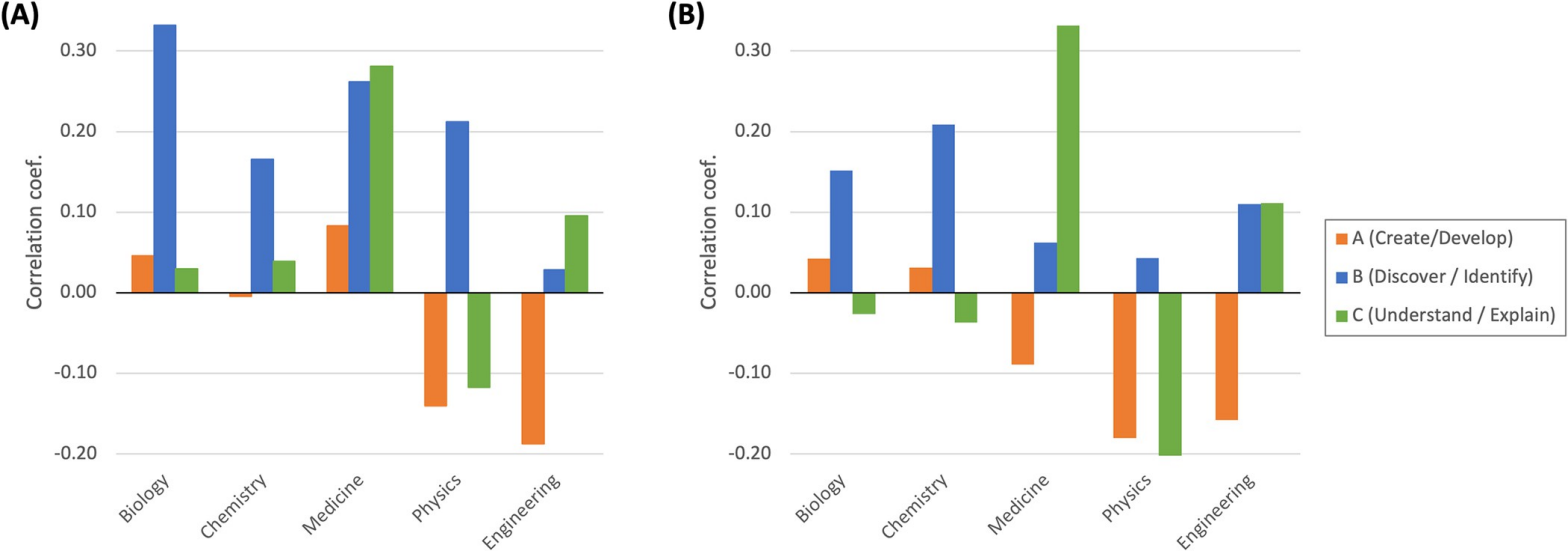
Field difference of word embedding. (A) Full model. (B) Field-specific model. Pearson’s correlation coefficients between the survey scores and *ElementNovel*_*10*_.

This field difference may be partly attributed to the difference in the performance of our word embedding model (Fig 1). Thus, we replicated the correlation analysis by using field-specific word embedding models (Fig 7). For easier comparison we used a 10-percentile value (*q* = 10) among the novelty measure series (*ElementNovel*_*10*_). Fig 7A is based on the word embedding trained by the full publication data (10-percentile values extracted from Fig 6), whereas Fig 7B is based on the word embedding trained by the text only in the focal field. The difference between the two panels (A and B) is rather subtle. We observed largely similar patterns for *Novel*_*A*_ ("creating and developing") and *Novel*_*C*_ ("understanding and explaining"). The correlation with *Novel*_*B*_ ("discovering and identifying") turns insignificant in medicine and physics, suggesting that the full model serves better to detect this novelty aspect. Thus, even though field-specific word embedding models may perform better in measuring document distances (Fig 1), we do not have a strong reason to use field-specific models in measuring novelty, especially when the boundary between scientific fields is becoming ambiguous [25, 26].

### 4.4 Comparing with existing novelty measures

Finally, we compare our novelty measures with two existing novelty measures. The first is an element novelty measure based on new words [23]. We assessed whether a scientific document contains any word that appears for the first time with the WoS publishing date. Many words first appeared in the early years of our dataset (1998–2018). To avoid detecting such spurious novelty, we set a baseline pool of words that appeared in the first five years (1998–2002). After excluding words in the baseline pool, we identified 1.4 million unique new words in 1.2 million documents published after 2003. We prepared a dummy variable coded 1 if a document contains at least one new word and 0 otherwise (*NewWord*).

The second is a recombinant novelty measure computed with the word embedding technique [16]. Their measure is based on the distance between cited references of a focal paper. Shibayama et al. [16] used an existing word embedding (*scispaCy*) to compute document vectors of all references, computed the cosine distance for all pairs of references, and took the maximum distance as the novelty measure. We employed this procedure with our word embedding model (*RecombNovel*).

**Fig 8:**
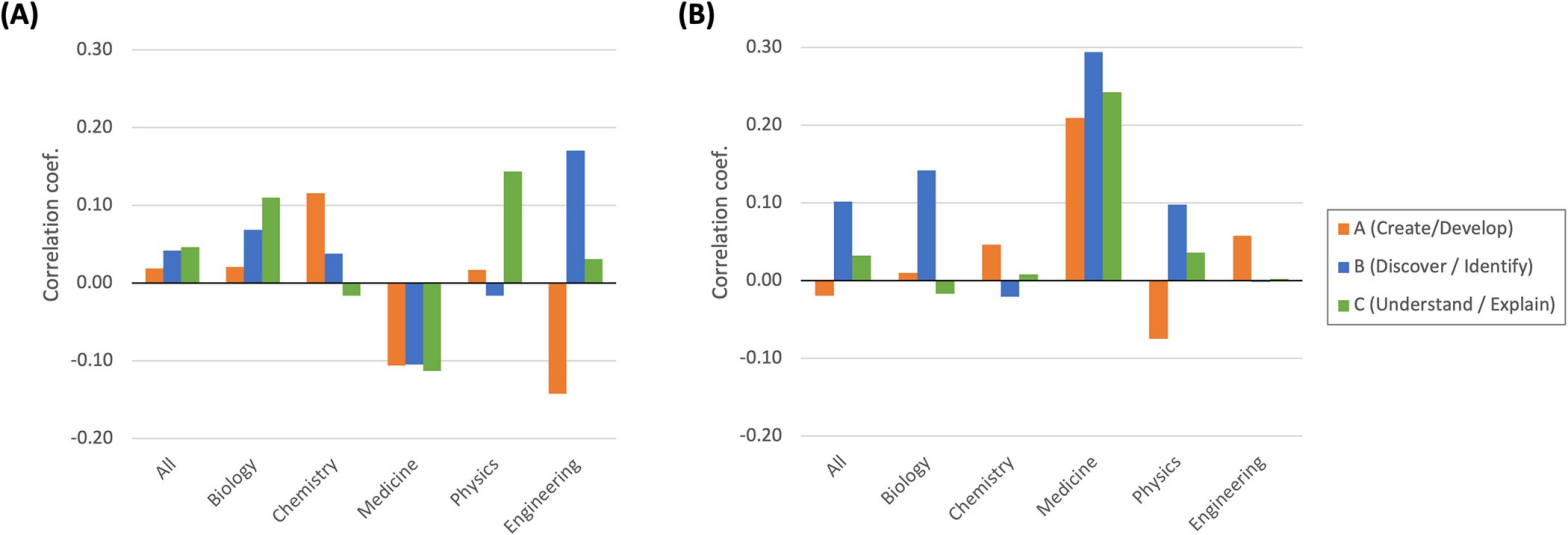
Correlation between existing novelty measures and survey scores. (A) *NewWord*. (B) *RecombNovel*.

Fig 8 presents the correlations between the existing novelty measures and the survey scores in all fields as well as in respective fields. Regarding the new-word element novelty, the correlations are rather small and insignificant overall (Fig 8A). Still, compared with our element novelty (Fig 7A), somewhat larger correlations are found for *Novel*_*A*_ ("creating and developing") in chemistry, *Novel*_*B*_ ("discovering and identifying") in engineering, and *Novel*_*C*_ ("understanding and explaining") in biology and physics. As to the recombinant novelty measure, the correlation with *Novel*_*B*_ ("discovering and identifying") turns rather modest except in medicine, and the correlation with *Novel*_*A*_ ("creating and developing") becomes stronger, though still modest, in chemistry, medicine, and engineering (Fig 8B).

## 5. Discussion

As novelty is a core value in science [1, 2] and technological progress [5, 6], several bibliometric measures for novelty have been proposed [12–16]. Nonetheless, a few fundamental limitations remain, which this study aims to address by offering a validated and field-universal approach to compute element novelty.

To this end, we first developed a word embedding model based on large-scale scientific text data. The word embedding technique draws on machine learning to extract semantic information from text data as high-dimensional vectors assigned to every word. We confirmed that word distances measured by our model are largely consistent with an established but field-specific word embedding model (*scispaCy*). We also found that document distances measured by our model are consistent with co-citation distances. Overall, these results give confidence to the performance of our word embedding model. A breakdown by scientific fields suggests that the performance of word embedding models varies across fields, and using a field-specific word embedding model may be suitable depending on the purpose.

Second, using the developed word embedding model, we computed element novelty measures. The validation with our survey scores suggests that the proposed novelty measure is strongly correlated with *Novel*_*B*_ ("discovering and identifying") across most scientific fields. This is consistent with our expectation in that discovering and identifying phenomena, substances, molecules tend to be represented by a new word or short expressions. We also computed element novelty with field-specific word embedding models without finding substantial improvement. Finally, compared with previous novelty measures, our element novelty measure is more strongly and stably correlated at least with *Novel*_*B*_. Therefore, our element novelty measure based on our own word embedding model seems to provide a strong approach to quantify element novelty in terms of discovering or identifying something new.

This study offers a few contributions. First, we provide a robust and versatile measure of element novelty. Despite its fundamental importance [23, 24], element novelty has been underserved in comparison to recombinant novelty [12, 14, 15, 20]. Our approach based on word embeddings is robust and efficient in comparison to existing measures of element novelty based on keywords [23]. Further, our approach is applicable irrespective of scientific fields. Some of the existing novelty measures are applicable only in certain fields [13, 14, 23], which is a critical flaw given increasing interdisciplinarity [25, 26].

Second, this study offers self-reported novelty data from multiple angles, with which previous and future bibliometric novelty measures can be tested. Previous novelty measures were often not validated. As novelty is a multifaceted concept [29, 30], it has been unclear what aspect of novelty is captured by previous measures. Our validation analyses clarify the novelty dimension that our measure captures as well as its differences from other bibliometric measures.

Third, this study presents a field-universal and validated word embedding model. It offers a technical basis to interpret scientific text information, which can be applied not only for calculating novelty of scientific articles but also for broader purposes. For example, the novelty score can be computed for any text information (e.g., funding applications). We can apply the word embedding to a collection of knowledge (e.g., research portfolio of individuals, organizations, etc.) and measure its qualities such as diversity. We can also apply it to text data over a certain time period and investigate the temporal evolution of knowledge.

This study nonetheless has a few limitations. First, we examined our word embedding model and the novelty measure in all fields except for social sciences and humanities. When science is becoming increasingly interdisciplinary, establishing a methodology applicable in these fields as well would be beneficial. Second, we need to further explore what aspect of novelty is captured by the novelty measure. Particularly, the validation analysis in different fields presents mixed results, and the interpretation is not always straightforward. Third, differences between various novelty indicators need further investigation. For example, we expected that our element novelty measure corresponds to *Novel*_*B*_ whereas previous recombinant novelty measures capture better new composite knowledge (*Novel*_*A*_ and *Novel*_*C*_), but the result is not perfectly clear.

## 6. Conclusions

This study offers a new bibliometric indicator of element novelty, drawing on a word embedding model trained on scientific publications in all disciplines, and validated it with self-reported novelty scores from our original survey. To further advance the methodology, a few directions of exploration are possible. First, the word embedding model can be further improved. Our main goal was to provide a validated and scalable method to measure element novelty, and we did not aim to optimize its performance once the validation analysis demonstrated satisfactory results. Nonetheless, the word embedding model can be improved with more advanced techniques such as Sentence-BERT or SciBERT [10, 50]. Also, the parameter setting of the machine learning can be fine-tuned, while we mostly followed the default setup. Further, we could expand the scope of the word embedding including documents from social sciences and humanities. Second, we can further explore correspondence between different aspects of novelty and various novelty indicators including ours. Our measure does not seem to capture a few aspects of novelty (*Novel*_*A*_ and *Novel*_*C*_). While this is desirable in that our measure has specificity, we need alternatives to measure these aspects. Recombinant novelty measures may serve the purpose, and future analyses should examine what novelty aspects are captured by various recombinant novelty operationalizations [12, 14, 15, 20]. We can also examine the correspondence in respective fields more thoroughly with larger validation data.

## Supporting information

S1 Fig Correlation between element novelty scores and survey scores. A series of element novelty scores (*q* = 0, 1, 5, 10, 15, and 20) are displayed. ^†^p<0.1. *p<0.05. **p<0.01.***p<0.001. As the survey scores of novelty, 3-point scale (1: not applicable– 2: improved– 3: novel) is used instead of the binary score.
(TIF)

S1 File Raw data of Fig 1. (CSV)

S2 File Raw data of Fig 2. (CSV)

S3 File Raw data of Figs 3–8. (CSV)
