# Supplementary figures and images for: Identify novel elements of knowledge with word embedding
Source: PLoS One. 2023 Jun 20;18(6):e0284567. doi: 10.1371/journal.pone.0284567 (PMC10281565; doi:10.1371/journal.pone.0284567)

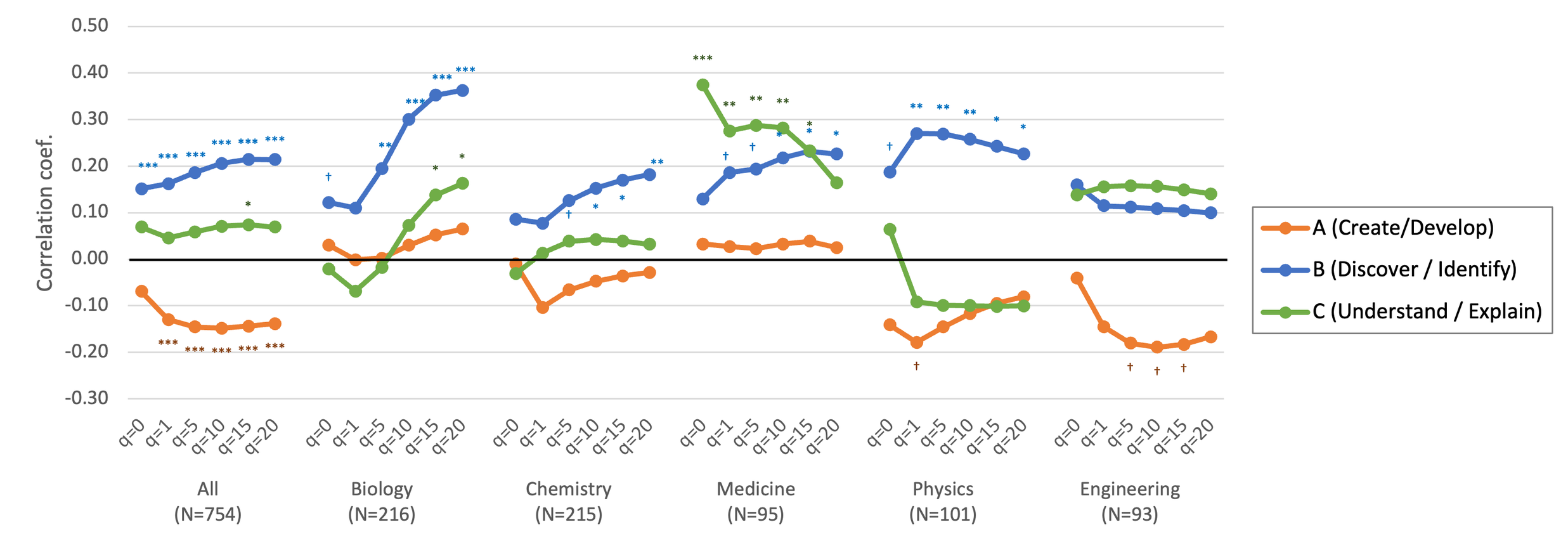

Supplement: S1 Fig — A series of element novelty scores (q = 0, 1, 5, 10, 15, and 20) are displayed. †p<0.1. *p<0.05. **p<0.01.***p<0.001. As the survey scores of novelty, 3-point scale (1: not applicable– 2: improved– 3: novel) is used instead of the binary score. (TIF) [file pone.0284567.s001.tif]
